# Postbiotics: emerging therapeutic approach in diabetic retinopathy

**DOI:** 10.3389/fmicb.2024.1359949

**Published:** 2024-03-04

**Authors:** Qin Chen, Xue-Jian Li, Wei Xie, Zhao-An Su, Guang-Ming Qin, Chen-Huan Yu

**Affiliations:** ^1^Eye Center, The Second Affiliated Hospital, School of Medicine, Zhejiang University, Hangzhou, China; ^2^Zhejiang Provincial Key Laboratory of Ophthalmology, Zhejiang Provincial Clinical Research Center for Eye Diseases, Zhejiang Provincial Engineering Institute on Eye Diseases, Hangzhou, China; ^3^Key Laboratory of Experimental Animal and Safety Evaluation, Hangzhou Medical College, Hangzhou, China; ^4^Animal Laboratory Center, Cancer Hospital of the University of Chinese Academy of Sciences (Zhejiang Cancer Hospital), Hangzhou, China; ^5^Institute of Basic Medicine and Cancer, Chinese Academy of Sciences, Hangzhou, China

**Keywords:** postbiotic, gut-retinal axis, short chain fatty acids, secondary bile acids, safety

## Abstract

Diabetic retinopathy (DR) is a prevalent microvascular complication in diabetic patients that poses a serious risk as it can cause substantial visual impairment and even vision loss. Due to the prolonged onset of DR, lengthy treatment duration, and limited therapeutic effectiveness, it is extremely important to find a new strategy for the treatment of DR. Postbiotic is an emerging dietary supplement which consists of the inactivate microbiota and its metabolites. Numerous animal experiments have demonstrated that intervention with postbiotics reduces hyperglycemia, attenuates retinal peripapillary and endothelial cell damage, improves retinal microcirculatory dysfunction, and consequently delays the progression of DR. More strikingly, unlike conventional probiotics and prebiotics, postbiotics with small molecules can directly colonize the intestinal epithelial cells, and exert heat-resistant, acid-resistant, and durable for storage. Despite few clinical significance, oral administration with postbiotics might become the effective management for the prevention and treatment of DR. In this review, we summarized the basic conception, classification, molecular mechanisms, and the advances in the therapeutic implications of postbiotics in the pathogenesis of DR. Postbiotics present great potential as a viable adjunctive therapy for DR.

## 1 Introduction

Diabetes mellitus (DM) is a chronic disease that is widely prevalent worldwide, with an extremely high incidence, leading to a variety of complications such as macrovascular or microvascular lesions. The number of adults with this disease has more than tripled in the last two decades. According to the IDF Diabetes Atlas, there are currently 537 million people living with diabetes globally, a number that is expected to rise to 784 million by 2045 (Sun et al., 2022). Diabetic retinopathy (DR) is the most common microvascular complication in DM patients with poor glycemic control. Its pathogenesis is complex, involving a variety of causative factors. Currently, it is believed that chronic hyperglycemia is the primary basis of its pathogenesis, leading to microvascular damage and retinal dysfunction (Song et al., 2021). It has been reported that approximately 35% of diabetic patients have varying degrees of retinopathy, with nearly 10% at risk of progressing to blindness (Lin et al., 2021). It is widely recognized that DR is the leading cause of DM-related visual impairment or blindness in working-age and elderly individuals worldwide (Teo et al., 2021). However, the underlying mechanisms of DR pathogenesis remain unclear, and there are no specific drugs for its treatment.

Recently, gut dysbiosis has been demonstrated to be one of the important causes of DR (Huang et al., 2021; Bai et al., 2022; Liu et al., 2022). Numerous studies have shown that the intestinal microbiota in the patients with type 2 diabetes mellitus (T2DM) is often altered; these changes can not only affect intestinal glucose metabolism, but also cause insulin resistance, inflammation, oxidative stress, vascular endothelial dysfunction, and other pathological damage by altering various pathways, potentially leading to serious ocular complications (Liu et al., 2022, 2023). Therefore, regulating gut microbiota has become a novel strategy for treating DR. Microecological agents, such as probiotics, prebiotics, and synbiotics, play a crucial role in maintaining the balance of gut microbiota and enhancing blood glucose levels. However, their use is limited during certain special circumstances, such as pregnancy, immunodeficiency, and severe infections.

Postbiotics are defined as “inactivated bacteria and bacterial components that have a beneficial effect on the host.” They include cellular components, secreted materials, metabolites, and non-viable microorganisms, which play the vital roles in restoring gut microbiota and alleviating microangiopathy. Given their unique biological activity and potential to replace antibiotics, postbiotics have been widely used in general food, health food, and gastrointestinal therapeutic drugs (Tsai et al., 2019). Especially, it can lower blood glucose levels, improve insulin sensitivity, and shorten DR duration. Therefore, non-viable postbiotics have recently been used as a better alternative for the treatment and prevention of metabolic diseases and their complications (Abdelazez et al., 2022; Mishra et al., 2023). This paper summarizes the various types of postbiotics and their potential benefits in preventing DR and highlights recent advancements in their clinical applications.

## 2 Gut dysbiosis and its relation to DR

**Figure 1:**
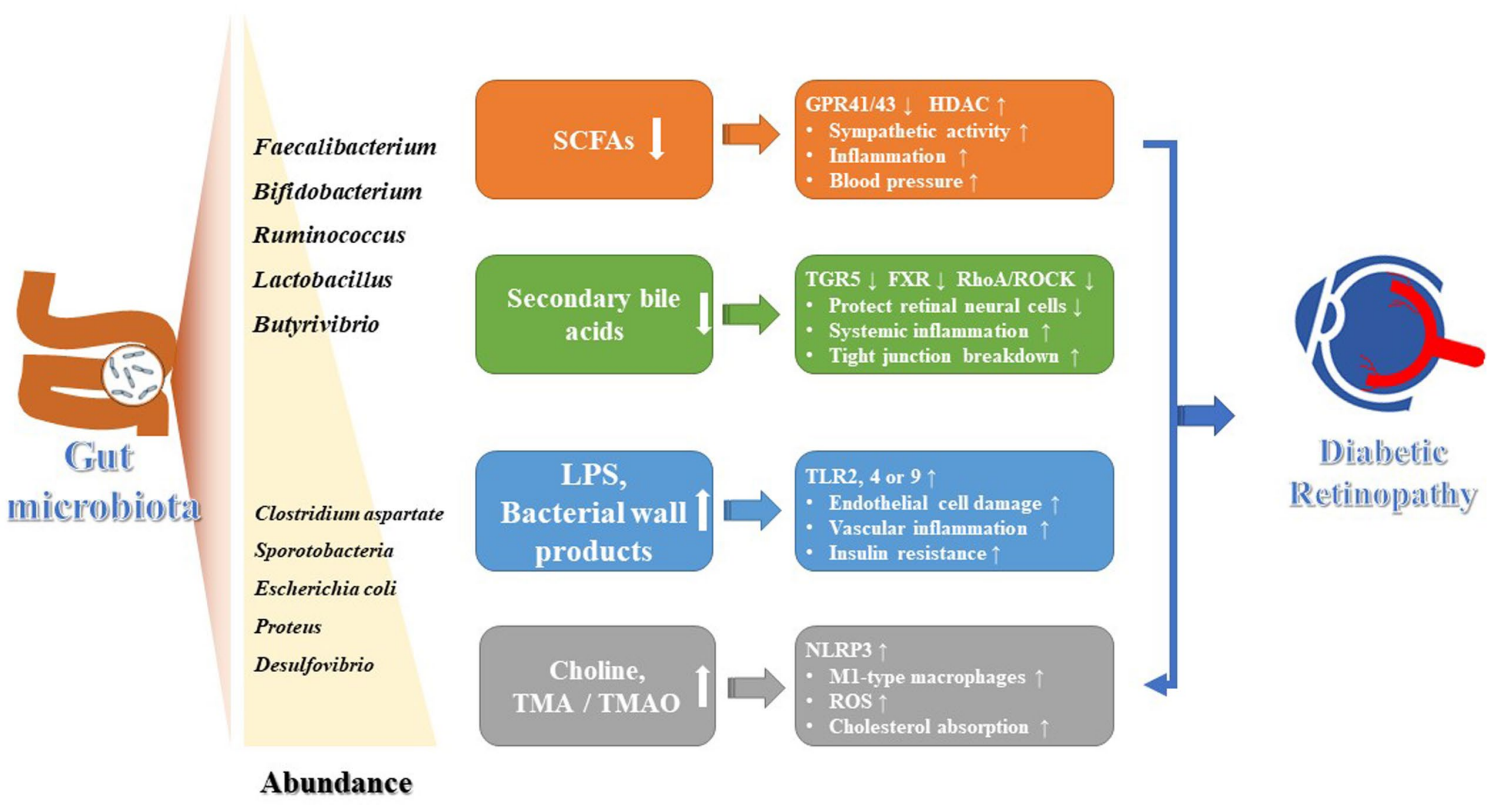
The roles of gut microbiota and its metabolites on the development of DR.

Gut dysbiosis is a common characteristic of patients with DR, leading to a remarkably reduction in the bacterial abundance and species (Figure 1). The gut microbiome of patients with DR was more distinct than that of patients with T2DM and healthy controls (HC). Compared with HC, there was a reduced abundance of anti-inflammatory genera such as *Roseburia*, *Lachnospira*, and *Blautia* in T2DM patients. In addition to those 3 genera, other genera like *Faecalibacterium*, *Bifidobacterium*, *Ruminococcus*, *Mitsuokella*, *Streptococcus*, *Lactobacillus*, and *Butyrivibrio* were also found to be decreased in DR patients (Das et al., 2021). The absence of these microbiota would reduce the production of organic acids, such as butyric acid and lactic acid, as well as the secretion of anti-inflammatory mediators, such as IL-10 and anti-inflammatory protein MAM, in intestine. This reduction affected the intestinal barrier function, leading to a systemic inflammatory response. In particular, elevated serum endotoxin directly accelerated damage to diabetic retinal endothelial cells and promoted the progression of DR (Tian et al., 2021; Huang et al., 2023). But in another clinic trail, four pro-inflammatory pathogens (*Aspergillus*, *Diutina*, *Pseudogymnoascus* and *Cladorrhinum*) were sharply decreased in DR patients compared with HC (Jayasudha et al., 2020). Additionally, gut dysbiosis could compromise the intestinal mucosal barrier by producing metabolic products like trimethylamine oxide (TMAO), lipopolysaccharides (LPS), choline, and amino acid metabolites (Liu et al., 2022; Xue et al., 2023). This can lead to increased intestinal permeability and temporary disruption of the blood-retinal barrier, allowing these substances to enter the eye and trigger host immune response, thereby contributing to the development and advancement of ocular diseases.

Although multiple mechanisms for changes in microvascular and glial components have been identified, the functional and structural mechanisms of cellular damage and cellular adaptive changes in the retina remain unknown due to the complex pathogenesis of DR. It had been reported that intermittent fasting alters gut microbiota composition and/or circulating bile acid levels, resulting in the increase of tauroursodeoxycholic acid (TUDCA), a secondary bile acid with neuroprotective properties. This bile acid subsequently prevented retinopathy by TGR5 activation in retinal neural cells (Haluzík and Mráz, 2018). However, there is not any direct association between these alterations and DR; instead, they are part of an intermediate process. Currently available studies have only emphasized the involvement of gut dysbiosis in the pathogenesis of T2DM. The connection between gut microbiota composition and DR is still far from being fully understood.

## 3 The conception and classification of postbiotics

Probiotics and synbiotics are commonly used as food supplements in the food industry to promote a balanced diet and enhance the health of the intestinal microbiota. However, with the gradual increase in research, more and more evidence has showed that the use of probiotics or synbiotics in the treatment of certain diseases has not achieved the expected effectiveness (Piqué et al., 2019). This is because probiotics need to colonize, compete for intestinal adherence, and balance the intestinal microbiota to promote good health. New scientific evidence indicates that probiotics are beneficial to the body’s health. It is not necessarily the live bacteria that have a direct relationship, but rather the metabolites or bacterial components of live bacteria that promote health. As a result, probiotics have been gradually recognized to exert beneficial effects based on their bacteria themselves, metabolites, or lysate products, which are formally referred to as postbiotics by International Scientific Association of Probiotics and Prebiotics (Salminen et al., 2021).

The definition emphasizes that postbiotics must be components (e.g., cell walls, lipoteichoic acid and exopolysaccharides) and metabolites (e.g., short-chain fatty acids, bacteriocins, tryptophan catabolites and vitamins) of inactive microorganisms that, in certain doses, can produce health benefits for the host. Their beneficial functions include, but are not limited to, antimicrobial and antioxidant properties, as well as modulation of intestinal barrier function and immune response. Much of the current postbiotic research is focused on *Lactobacillus* and *Bifidobacterium*, similar to probiotic organisms (Magryś and Pawlik, 2023; Motei et al., 2023). Overall, postbiotics offer advantages that probiotics cannot match, including a well-defined chemical structure, safe dosage parameters, and a longer shelf life of up to 5 years as a nutritional additive. Furthermore, postbiotics are effectively absorbed, metabolized, and distributed throughout the body. The use of postbiotics can provide probiotic-like benefits while avoiding issues such as low bioavailability of live bacteria, unstable effects, and the potential for resistance gene transmission (Suthar et al., 2023). This will mark a new direction for future research in the field of probiotics.

## 4 Function and application of postbiotics in DR treatment

**Figure 2:**
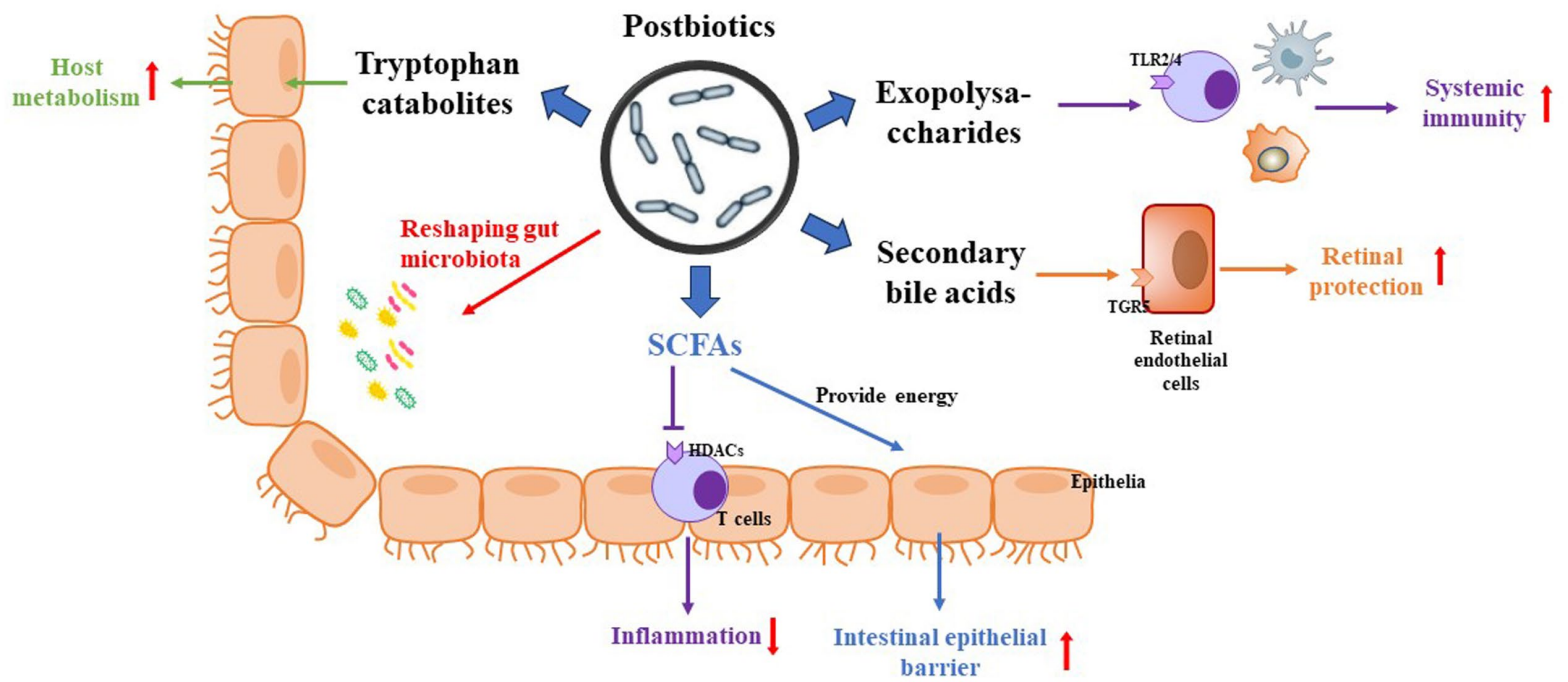
Potential mechanism of postbiotics on DR. (1) Postbiotics reshape the populations of gut microbiota. (2) SFCAs not only provide calories for the host but also inhibit HDACs via activation of inflammatory signaling. (3) Exopolysaccharides regulate host immunity via activation of TLR2/4 in immune cells. (4) Secondary bile acids regulate host metabolism and present neuroprotection via activation of TGR5. (5) Tryptophan catabolites regulate host metabolism and inhibit inflammation.

Postbiotics can regulate body metabolism through systemic reactions *in vivo* and *in situ* reactions in the intestinal lumen. *In vivo* systemic reaction refers to the ability of postbiotics to act more rapidly and directly on the human body after heat inactivation, thanks to their small molecule characteristics. This allows them to enhance human immunity, balance intestinal microbiota, and regulate physiological functions by crossing the intestinal barrier. The luminal *in situ* reaction refers to the ability of compounds like fatty acids to inhibit the growth of harmful bacteria in the intestinal lumen, decrease the aggregation of harmful bacteria, and maintain a balanced ratio of beneficial and harmful bacteria in the intestinal tract (Mosca et al., 2022). This process contributes to the overall health of the human intestine. Thus, the health benefits offered by postbiotics may be driven by various mechanisms (Figure 2). Recently, scientists have identified various forms of postbiotics. The postbiotic components produced by probiotic cells can beneficially regulate the body’s metabolism through different pathways. Some postbiotics exhibit probiotic effects like those of probiotics, and in some cases, their mechanisms may also be like the known probiotic mechanisms. Notably, postbiotics are not reliant on bacterial activity, and the probiotic mechanisms of these postbiotics can also function independently or in combination (Salminen et al., 2021). However, up to now, the mechanism of action of postbiotics for their beneficial effects in humans or animals has not been fully understood.

### 4.1 Short-chain fatty acids (SCFAs)

SCFAs are metabolites produced by intestinal microbiota that have significant importance for the human body. When dietary fiber is fermented by intestinal microbiota, SCFAs like acetic acid, propionic acid, and butyric acid are generated. Some SCFAs enter the bloodstream through the portal vein, while others serve as an energy source for the epithelial cells of the colonic mucosa, providing calories for the host (Fang et al., 2022). SCFAs play a crucial role in mitochondrial energy metabolism, as well as regulating glucose and lipid metabolism, immune responses, and inflammation levels. They exert their biological functions through two main pathways: binding to free fatty acid receptors (FFAR2/FFAR3) as ligands, and inhibiting histone deacetylases (HDACs) to regulate gene expression (Mirzaei et al., 2021; Zhang et al., 2022b). However, the effects of SCFAs on host function remain inconsistent due to their complex biological functions, especially when interacting with signaling pathways in the host. In terms of glucose metabolism, high-fat foods rich in butyrate have been found to promote thermogenesis and energy expenditure in mice, while acetate injections in rats improved their glucose tolerance capacity. SCFAs can also cross the blood–brain barrier and play a role in the central nervous system, potentially entering the retina through the retinal inner blood-retinal barrier (iBRB) and regulating inflammation and oxidative damage (Schaefer et al., 2022). Additionally, as SCFAs act as inhibitors of HDACs, they may play a role in regulating the development of DR through epigenetic pathways, given the multiple ways in which epigenetically related histones are modified in DR disease development (Tawarayama et al., 2023).

### 4.2 Secondary bile acids (SBAs)

Primary bile acids, derived from cholesterol and combined with taurine or glycine in the liver, are stored in the gallbladder and released into the duodenum during feeding to aid in the emulsification of dietary lipids. While most of these primary bile acids are reabsorbed in the intestine, a small percentage is degraded by anaerobic bacteria in the gut, resulting in the production of secondary bile acids such as TUDCA and ursodeoxycholic acid (UDCA) (Grüner and Mattner, 2021). These hydrophobic secondary bile acids are mainly excreted in feces, with a small portion entering circulation.

Secondary bile acids, known as active metabolites, possess bioregulatory activity and act as signaling molecules within the human body. They play a role in regulating host metabolism by binding to the nuclear receptor FXR and the G-coupled membrane protein 5 (TGR5) receptor (Win et al., 2021). Study has displayed that intermittent fasting prevents diabetic retinopathy in db/db diabetic mice by reshaping the microbiota to favor species that produce TUDCA, leading to subsequent retinal protection through TGR5 activation (Beli et al., 2018). As a result, secondary bile acids, such as TUDCA, contribute to reducing the incidence of DR. TUDCA, a neuroprotective BA, has been found to decrease the levels of nitric oxide (NO) and down-regulate the expression of proteins such as ICAM-1, NOS, NF-κB, and VEGF, thereby slowing down the progression of DR (Fu et al., 2021; Lenin et al., 2023). Similarly, UDCA has shown the ability to reduce retinal inflammation in a mouse model of STZ-induced diabetes (Ouyang et al., 2018). It achieves this by attenuating endoplasmic reticulum stress-associated peripapillary retinal cell loss through the inhibition of ionized calcium-binding adapter molecule 1 (Iba-1) expression (Chung et al., 2017). It is important to note that a weakened bile acid signaling pathway can exacerbate DR pathology, while an upregulated or activated TGR5 pathway can slow down its progression by inhibiting RhoA/ROCK and PKCδ/Drp1-HK2 pathways (Zhu et al., 2020; Zhang et al., 2022a).

### 4.3 Exopolysaccharides (EPSs)

EPSs are carbohydrate polymers found on the surface of most bacteria in the form of pods or pericellular mucus. Recently, EPSs had a wide range of applications in food and medical practice, due to their distinguished immunomodulatory functions, including preventing the formation of bacterial biofilms and maintaining the balance of the intestinal microenvironment (Zhang et al., 2023). EPS derived from *Lactobacillus paracasei* DG exhibited immunostimulatory properties by upregulating the expression of TNF-α, IL-6, IL-8 and CCL20 genes in the human monocyte THP-1 cells (Balzaretti et al., 2017). Additionally, EPSs regulate innate and adaptive immune responses, stimulating T cells, B cells, NK cells, and macrophages to eliminate pathogen and scavenge free radicals (Rusinova-Videva et al., 2022; Niu et al., 2023). The polysaccharide produced by *Bacillus fragilis* can bind to the TLR2 receptor, upregulate regulatory T cells, inhibit the production of IL-17, and promote the expression of IL-10, thereby inhibiting inflammation (Sittipo et al., 2018). An EPS from *Bacillus subtilus* reduced the serum levels of intercellular adhesion molecule (ICAM), and vascular cell adhesion molecule (VCAM) levels in STZ-induced diabetic rats, and improved hyperglycemia- microvascular endothelial cell injury (Ghoneim et al., 2016).

### 4.4 Amino acid metabolites

Gut microbiota can produce a variety of amino acids and their intermediates, which protect against high glucose-induced microvascular endothelial damage and are the rate-limiting materials for glutathione synthesis (Mardinoglu et al., 2015; Beaumont and Blachier, 2020). A 12-year follow-up study by metabolomics analysis showed that N-lactoyl isoleucine, N-lactoyl valine, N-lactoyl tyrosine, and N-lactoyl phenylalanine, N-(2-furoyl) glycine, and 5-hydroxylysine were associated with an increased risk of DR, while citrulline was associated with a decreased risk of DR (Fernandes Silva et al., 2023). Increased oxidative stress in the retinal iBRB is partly due to the production of reduced glutathione.

Notably, among the amino acids studied, tryptophan is an essential amino acid, which can be metabolized primarily to kynurenine by indoleamine 2,2-dioxygenase of *Lactobacillus reuteri* and *Clostridium sporogenes*. The tryptophan derivative indole activates the aromatic hydrocarbon receptor of CD4^+^ T cells in the mouse intestine, induces their differentiation into CD4^+^CD8αα^+^ double-positive intraepithelial lymphocytes, which would promote the secretion of IL-22 and IL-10, and then inhibit inflammatory responses (Cervantes-Barragan et al., 2017). Clinical trial demonstrated that the level of plasma tryptophan was significantly decreased whereas kynurenine was increased in patients and mice with DR, indicating that the altered tryptophan–kynurenine metabolism pathway plays a key role in the pathogenesis of DR (Wang et al., 2022). Moreover, kynurenic acid, the final catalysate of kynurenine, serves as anti-inflammatory mediator and can cross various endothelial barriers to access the central and peripheral nervous systems, including the visual nervous system. Thus, kynurenic acid administration potentially contributes to the treatment of retinal aging and neurodegeneration (Fiedorowicz et al., 2019).

On the other hand, intestinal microbiota can modulate neuroendocrine and intestinal immune responses by regulating tryptophan metabolism, leading to the production of serotonin, kynurenine, tryptophan, indole, and their derivatives (Yano et al., 2015). Among these, serotonin is a crucial monoamine neurotransmitter that regulates central neurotransmission and intestinal physiological functions. Given that serotonin levels in patients with proliferative diabetic retinopathy (PDR) are significantly lower than in healthy subjects, and that the incidence of DR is lower in diabetic patients taking serotonin reuptake inhibitors compared to controls, it is possible that gut microbiota influences DR by regulating the production of tryptophan and serotonin (Kern et al., 2021).

### 4.5 Other metabolites

Gut microbiota can synthesize a variety of small molecules with signaling effects, such as hydrogen sulfide, which influences the development of DR. It was found that the levels of hydrogen sulfide in the vitreous and plasma of patients with PDR was significantly higher than those in the healthy individuals (Han et al., 2020). Administration with exogenous hydrogen sulfide also protects the retina by reducing oxidative stress in streptozotocin-induced diabetic DR rats (Si et al., 2013). Furthermore, different postbiotic fractions from *Lactobacillus rhamnosus* GG exert significant immunomodulatory effects via inactivation of TLR4/7, MAPK, ERK, and NF-κB signaling pathways in LPS-stimulated mouse RAW264.7 cells (Qi et al., 2020).

Extracellular vesicles (EVs) derived from gut microbiota mediate the communication between microorganisms and their host to the maintenance of intestinal homeostasis and may ultimately be implicated in the regulation of various metabolic diseases (Chen et al., 2022; Díez-Sainz et al., 2022). Several studies have demonstrated a direct relationship among EVs and gut barrier integrity and metabolic status in high fat diet-induced diabetics mice; oral administration of EVs from *Akkermansia muciniphila* (AKK) could reduce gut barrier permeability and improve glucose tolerance in diabetic mice (Chelakkot et al., 2018; Nah et al., 2019; Moosavi et al., 2020). But the roles of EVs on high glucose-induced DR should be investigated by more experiment and clinical verification.

## 5 Security issues

Compared to conventional probiotics and prebiotics, postbiotics as novel food supplements can directly colonize the intestinal epithelial cells for controlling the microbial population, and exert heat-resistant, acid-resistant, and durable for storage. Several clinical studies have investigated the potential for absorption, metabolism, and distribution of postbiotics (Kim et al., 2023; Sato et al., 2023). The benefits of postbiotics, including their defined chemical structure, low toxicity, minimal storage requirements, long shelf-life, and stability, offer significant advantages in microbe-related products. However, in a randomized controlled trial involving 40 children under 5 years of age, 36 of 40 children experienced relief from symptoms, and one child experienced severe dehydration (Malagón-Rojas et al., 2020), suggesting the potential adverse effects of postbiotics. Therefore, further studies should prioritize enhancing the safety of postbiotics during use and minimizing adverse reactions.

## 6 Future challenges and prospects

As byproducts of the intestinal microbiota, postbiotics can serve a wide range of functions in the intestinal tract and throughout the body. Up to now, the anti-inflammatory, antimicrobial, antitumor, hypoglycemic, and hypolipidemic effects of postbiotics have been substantiated. These effects have led to the manufacturing and marketing of postbiotics as health care products for the prevention and treatment of diseases. Recently, the most common application is still adding postbiotic elements to dairy products to boost immunity and regulate intestinal function. Especially in livestock feed supplementation, it can replace the use of some antibiotics, demonstrating its significant practical value. Emerging studies have confirmed the potential benefits of postbiotics in treating and alleviating obesity and diabetes (Osman et al., 2021; Balaguer et al., 2022). The main strains involved are AKK, Bifidobacterium, and Lactobacillus. The diversification of postbiotics in various forms indicates a promising future for their development in preventing and treating metabolic diseases. However, there is still a gap in its clinical applicability, long-term and short-term toxicity, and bioavailability, which are urgent issues for current research.

In addition to preventing and treating certain diseases, some of the characteristics of postbiotics also offer clear advantages compared to probiotics. As mentioned earlier, they can mitigate some of the health risks associated with probiotics. Additionally, postbiotics offer greater convenience in terms of storage and transportation. However, there are still many limitations in the current research on postbiotics. Firstly, there is a lack of understanding regarding how various metabolites or bacterial components interact with intestinal cells and influence downstream pathways. Secondly, the relationship between bacterial components and their effects, as well as the conformational relationship resulting from the combination of different types of postbiotics, remains unclear. Thirdly, short-chain fatty acids can be obtained through the fermentation of plant polysaccharides by intestinal microorganisms, extraction of cell-free supernatant via centrifugation and filtration, and production of bacterial lysates through chemical or mechanical degradation. As a result, there is still no unified standard and process for the mass production of postbiotics technology. Additionally, safety and regulatory concerns must be addressed, and consistent regulatory standards need to be established. Despite its faults, the potential use of postbiotics in medical and healthcare fields deserves attention. Furthermore, it is worth exploring the combination of postbiotics and prebiotics, as well as the potential application value of different postbiotic combinations.

## Author contributions

QC: Writing – original draft, Writing – review & editing. X-JL: Conceptualization, Writing – original draft. WX: Conceptualization, Writing – original draft. Z-AS: Visualization, Writing – review & editing. G-MQ: Visualization, Writing – review & editing. C-HY: Funding acquisition, Project administration, Supervision, Writing – review & editing.
